# COVID-19-Related Risk, Resilience, and Mental Health Among Mexican American Mothers Across the First Year of the Pandemic

**DOI:** 10.1007/s40615-023-01849-2

**Published:** 2023-11-08

**Authors:** Amy L. Non, Elizabeth S. Clausing, Sandraluz Lara-Cinisomo, Kimberly L. D’Anna Hernandez

**Affiliations:** 1 Department of Anthropology, University of California San Diego, La Jolla, CA USA; 2 Department of Anthropology, School of Global Integrative Studies, University of Nebraska, Lincoln, NE USA; 3 Center for Brain, Biology, and Behavior, University of Nebraska, Lincoln, NE USA; 4 Department of Kinesiology and Community Health, University of Illinois Urbana-Champaign, Urbana, IL USA; 5 Department of Psychology, Marquette University, Milwaukee, WI USA

**Keywords:** Latina, COVID-19, Stigma, Depression, Anxiety

## Abstract

**Background:**

Latina mothers have been especially affected by the pandemic and historically exhibit high rates of depression and anxiety. However, few longitudinal studies have assessed the effect of the pandemic on this vulnerable population. We hypothesized that COVID-19-related stressors would associate with psychological distress among Latina mothers across the first year of the pandemic.

**Methods:**

We investigated COVID-19-related impact, stigma, and fears across two critical time points and changes in these measures in relation to changes in maternal anxiety and depression among mothers of Mexican descent living in Southern California (*n*=152). Surveys were administered within 5–16 weeks of the March 19, 2020 stay-at-home COVID-19 order in California and again between June to December 2021.

**Results:**

High proportions of women reported moderate to severe impacts of COVID-19 early in the pandemic, which reduced modestly a year later, e.g., reduced family incomes (55.9% 2020 Lockdown vs 32.7% 1-year follow-up). Anticipatory stigma was high across the first year, e.g., worrying at least some of the time that a family member will be deported (33.1% 2020 Lockdown vs. 14.1% 1-year follow-up), or they would not be able to care for their children (88.5% 2020 lockdown vs 82.2% 1-year follow-up). COVID-19 stigma, impact, and fears were significantly associated with higher levels of anxiety and depressive symptoms at both time points (*p*<0.003), and changes in COVID-19 impact were associated with changes in depression (*p*=0.0004).

**Conclusion:**

Findings emphasize the adverse socioeconomic and psychological effects of the pandemic for Latina mothers.

**Supplementary Information:**

The online version contains supplementary material available at 10.1007/s40615-023-01849-2.

## Introduction

The coronavirus disease of 2019 (COVID-19) has affected every aspect of daily life since the World Health Organization declared it a pandemic [1]. While research has focused on higher hospitalizations and death rates among marginalized communities [2, 3], the psychosocial effects of the pandemic have received comparatively less attention. Social distancing, remote work, school closures, loss of jobs, and fears of infection have contributed to an increased stress burden. These dramatic changes to daily life began in California on March 19, 2020, when the statewide “shelter-in-place” order was imposed. For 2 months, all non-essential services shut down, massive layoffs occurred, and residents were told to only leave home for necessities [4]. Individuals across the USA felt these changes, but for several reasons detailed below, Latina women were especially affected, particularly early in the pandemic [5].

Latina women were at especially high risk for socioeconomic distress related to the pandemic, due to high rates of job loss, particularly in sectors hard hit by pandemic shutdowns such as hotels, restaurants, retail, and education, which disproportionately employ many Latina and Black women [6, 7]. In fact, Latina women suffered the highest rate of job loss early in the pandemic, with a fourfold increase in unemployment rates between February and April of 2020 [6], such that Latina women experienced the highest unemployment rate of all groups when shutdowns began in April 2020 [8]. Many Latina women were also deemed essential workers who retained their jobs, but received reduced earnings relative to non-Latina White Americans [9], and were less able to work from home [10]. While economic conditions improved as lockdowns were lifted, young Latina workers did not recover as quickly as other groups, with 4% fewer employed by May 2021, relative to before the pandemic [8].

We draw on cultural stress theory to understand why Latina mothers were especially at high risk of worsening mental health in the pandemic. Cultural stress theory posits that post-migration-related cultural stressors can increase stress among parents and their children and compromise family functioning [11, 12]. While not all Latinx in the USA are immigrants, they all are affected by decades of systemic racism in the USA, including constrained economic opportunities to low-wage and unstable work with less access to healthcare [13, 14]. Latinx people are also at risk of increased pandemic-related stigma as low-income — and especially immigrants and other marginalized groups — which have historically been scapegoated for spreading infectious diseases, such as HIV/AIDS, Ebola, and H1N1 [15, 16]. Latina immigrant mothers also face additional fears of having their families be separated by deportation, which restricted women from seeking COVID-19 testing or treatment [17]. However, little research has examined how these cultural stressors may have affected mental health of Latina women during the pandemic.

In addition to the unique cultural stressors faced by Latina women during the pandemic, mothers in general also faced the additional burdens of increased childcare and homeschooling duties [18] and high levels of emotional labor of caring for the entire family [19]. Women in general report much higher rates of disrupted lives due to the pandemic, such as sheltering in place more often than men [20]. Maternal fear of infection, especially for their children, may have contributed to decreased mental health during the pandemic, though this has not yet been studied among Latina women. These combined sociocultural stressors may have exacerbated risks of anxiety and depression for Latina mothers during the pandemic [21]. Less studied is whether these stressors have had lasting mental health effects beyond the initial lockdown period.

Latina women have historically experienced a heightened risk of recurrent depression relative to non-Latina White Americans [22], which has potentially worsened during the pandemic [23]. To examine the well-being of Latina mothers across the pandemic, we describe the psychosocial impact (socioeconomic changes and stressors) of COVID-19 among mothers of Mexican descent living in Southern California, at two time points across the first year of the pandemic: within a few months of the first nationwide lockdowns, and just over a year later. We compared levels of pandemic-related stressors across this period, including psychological and social impacts of COVID-19 (e.g., socioeconomic, family, healthcare), anticipatory stigma, and COVID-19-related fears. Finally, we examined the effect of these pandemic-related stressors on Latina women’s mental health at both time points and how change in these stressors predicted change in mental health over time. We hypothesized that COVID-19-related stressors would negatively affect the mental health of Mexican-descent mothers, and effects would be lasting across the first year of the pandemic.

## Methods

Our study utilizes a cohort of Latina women of Mexican descent living near the US–Mexico border in Southern California [24]. Beginning in 2013 and continuing up to 2019, pregnant women were recruited from a local community clinic, and followed up to the 2020 COVID-19 pandemic. At the time of recruitment in the initial study, participants were of Mexican descent, 18 years or older, less than 15 weeks pregnant, with no current illicit substance or tobacco use, carrying a singleton pregnancy (not multiple fetuses), and were not on any medications. Surveys were conducted in participants’ preferred language (English or Spanish). This study is based on 152 women (all who responded out of 309 contacted from the original cohort) who were longitudinally surveyed within 5–16 weeks of the March 19, 2020, stay-at-home order in California (April–July 2020), hereafter referred to as the 2020 lockdown period, and then again 15–21 months after the phase I shutdown (June–December 2021), hereafter labeled as 1-year follow-up. The study was performed in accordance with the ethical standards of the 1964 Declaration of Helsinki and its later amendments. Informed written consent was provided by all participants, and all procedures were approved by the Institutional Review Board at California State University San Marcos and Marquette University.

### Measures

#### Demographics

Participants self-reported all demographic characteristics and COVID-19-related changes to socioeconomic variables. Education and parity were measured at baseline only, while the remaining demographics were also measured during the early months of the pandemic. Acculturation was measured with the Short Acculturation Scale for Hispanics (SASH) [25], using the mean of 12 items (ranging 1–5). Higher scores indicate more acculturation. The SASH scale had high internal consistency with Cronbach’s alpha=0.93.﻿﻿

#### COVID-19-Related Stressors

COVID-19 impact was measured with the coronavirus impact scale [26, 27], containing nine questions including pandemic impact on routines, family income, food access, medical and mental healthcare access, access to social support, experiences of stress related to the pandemic, discord in the family, and personal diagnosis/symptoms of COVID-19 (see S1 table for list of questions). A COVID-19 impact score was created as the mean of responses to eight questions (ranging from 0=no change to 3=severe change) with higher values indicating greater impact. See Supp. Table 1 for list of questions. Cronbach’s alpha was 0.78 at the 2020 lockdown period.

Anticipatory COVID-19-related stigma was assessed with a 10-item stigma/discrimination scale (see S2 Table). Four questions were drawn from an HIV/AIDS stigma scale [28], and six were created, similar to a migration experience scale [29]. The mean response of all questions (ranging 1–4) was used, with higher scores indicating more anticipatory stigma. See Supp. Table 2 for a list of questions. The scale had high internal consistency with Cronbach’s alpha=0.90 at the 2020 lockdown period.

Fears related to COVID-19 were assessed using a modified Fear of Illness and Virus Evaluation (FIVE) scale [30], shortened and validated in Spanish [31]. We used an abbreviated 19-item version, which included two of the four subscales: (a) fears about contamination and illness (9 questions) and (b) fears about social distancing (10 questions). The questions ranged in response from 1=not worried at all to 4=worried all of the time. The fears about contamination scale had high internal consistency with Cronbach’s alpha=0.94 at 2020 Lockdown. The fears about social distancing had a Cronbach’s alpha=0.76 at 2020 lockdown. The means of responses (ranging 1–4) in each subscale were used, with higher scores indicating greater levels of fear. See S3 Table for list of all fear-related questions.

#### Mental Health Measures

The Center for Epidemiologic Studies Depression Scale (CES-D) assessed depressive symptoms in the last week [32], using the sum of responses (ranging 0–3) to 10 items. The State-Trait Anxiety Inventory (STAI – Form Y-1) measured participants’ state anxiety [33], as the sum of responses to 6 items (ranging 1–4). Higher scores indicate more anxiety symptoms. The Cronbach’s alpha for CES-D was 0.79 and for STAI was 0.77 at the 2020 lockdown period.

### Data Analysis

Paired Wilcoxon signed rank tests compared levels of descriptive statistics for continuous variables and paired McNemar tests for discrete variables from both time points. Bivariate correlations among all primary study variables were analyzed at each time point and in relation to change over time in primary variables. Associations between each COVID-19-related stressor and mental health outcome were each analyzed separately with linear regressions, all models adjusted for maternal age, relationship status, education, and acculturation. Shapiro-Wilk normality tests and visual inspection of Q-Q plots were used to test normality of the residuals of each model. Because residuals of the models of depression at both time points were positively skewed, we tried first a log and then a square root transformation, but only a Box-Cox transformation of CESD-D (after adding 1 to the CES-D score) created models with normally distributed residuals. Change scores in COVID-19 stigma, impact, and fear scales were calculated as levels of these measures at 1-year follow-up minus levels at the 2020 lockdown period, such that positive change scores in COVID-19 stressors indicate increases over time. Correlation and regression analyses were all Bonferroni adjusted.

## Results

Sample sociodemographics, health, and psychosocial factors at both time points are presented in Table 1. At the beginning of the pandemic, participants were primarily Mexican-born mothers (69.7%) of mean age 32.0 years, lived in the USA on average 20.2 years, mostly married (76.2%), with an average of 11.4 years of education, 32.9% employed, and 8.6% were pregnant. Over a year into the pandemic, significantly more women were employed relative to early in the pandemic (53.3% at 1-year follow-up vs. 32.9% at 2020 lockdown), and more were employed full time relative to early in the pandemic (66.7% vs. 42.0%), though annual household incomes were similar over time. The mean acculturation level was moderate (mean 2.2 on a scale of 1–5) and was only measured during the 2020 lockdown period.

**Table 1:** Sociodemographic and health measures of participants **(*n*=152)**

Sociodemographic variables	2020 lockdown period April–July 2020 *n*=152	1-year follow-up June–December 2021 *n*=152	*p*-value
	*n* (%) or mean (SD, range)	*n* (%) or mean (SD, range)	
Age	32.0 (6.09, 18–50)	33.3 (6.1, 19–51)	**<0.0001**
Married/partnered	115/151 (76.2%)	112/151 (74.2%)	
Mother’s birthplace			--
USA	43/152 (28.3%)	--	
Mexico/outside of the USA	106/152 (69.7%)	--	
Unknown	3/152 (2.0%)	--	
Years in the USA	20.2 (7.9, 1–46)	--	--
Years of education at baseline	11.4 (2.96, 3–18)	--	--
Household members	5.3 (1.7, 2–13)	5.2 (1.7, 2–14)	0.1175
Employed (includes part-time employment)	50/152 (32.9%)	81/152 (53.3%)	**0.0060**
Full time	21/50 (42.0%)	54/81 (66.7%)	
Part time	29/50 (58.0%)	27/81 (33.3%)	
Annual household income			
≤$4,999	25/149 (16.8%)	20/152 (13.2%)	0.4042
$5,000-$24,999	57/149 (38.3%)	54/152 (35.5%)	0.3711
$25,000-$49,999	49/149 (32.9%)	60/152 (39.5%)	0.1048
≥$50,000	18/149 (12.1%)	18/152 (11.8%)	--
Currently pregnant	13/152 (8.6%)	9/152 (5.9%)	0.5224
Parity at baseline			--
0	39/147 (26.5%)		
1	38/147 (25.9%)		
2	38/147 (25.9%)		
3+	32/147 (21.7%)		
Acculturation	2.24 (0.85, 1–4.5)	--	--
Health-related variables			
Personal COVID-19 diagnosis			
Tested positive	1/152 (0.7%)	43/152 (28.3%)	**<0.0001**
Ever took a test for COVID	3/152 (2.0%)	109/152 (71.7%)	**<0.0001**
Household member diagnosed with COVID-19			
Tested positive	1/152 (0.7%)	50/112 (44.6%)	**<0.0001**
Ever took a test for COVID-19	3/152 (2.0%)	112/152 (73.7%)	**<0.0001**
At least one immediate family member deceased from COVID-19, *n* (%)	1/152 (0.7%)	4/149 (2.7%)	0.3711
Lost family/close friend due to COVID-19	4/152 (2.6%)	43/150 (28.7%)	**<0.0001**
Depressive symptoms (CES-D)	6.17 (4.5, 0–22)	5.73 (4.8, 0–21)	0.2374
Anxiety (STAI)	12.1 (3.5, 6–24)	11.0 (3.8, 6–23)	**0.0030**

Sample size for all variables was *n*=192 unless otherwise specified. *P*-values are from paired Wilcoxon signed-rank tests for continuous variables and McNemar paired tests for discrete variables. Bold *p*-values indicate significant differences over time

### Socioeconomic Changes Due to COVID-19

At the 2020 lockdown period, a high proportion of women reported adverse socioeconomic effects due to COVID-19, including 23% reporting their household’s sole income earner lost their job, 39.6% were unemployed due to COVID-19, and 59.8% reported a partner working fewer hours (Table 2). At 1-year follow-up, the socioeconomic impact was smaller; e.g., more households had at least one member working outside the home (98.1% at 1-year follow-up vs. 88.0% at 2020 lockdown), and fewer employed women were only working part time (33.3% at 1-year follow-up vs. 72.0% at 2020 lockdown).

**Table 2:** Economic impact of COVID-19 and COVID-19-related stress scales

Economic impact variables	Frequency (%) 2020 lockdown period (April–July 2020)	Frequency (%) 1-year follow-up (June–December 2021)	*p*-value
Has the primary/sole income earner of your household lost their job?	35/152 (23.0%)	39/152 (25.7%)	0.6353
Unemployed due to COVID-19	40/101 (39.6%)	--	--
Is your partner working fewer hours than they did before quarantine?	73/122 (59.8%)	41/152 (27.0%)	**<0.0001**
Household members working outside the home: (range 0–7)			
0	17/152 (11.2%)	3/152 (2.0%)	**0.0022**
1	81/152 (53.3%)	71/152 (46.7%)	0.1748
2	28/152 (18.4%)	46/152 (30.3%)	**0.0087**
3+	26/152 (17.1%)	32/152 (21.1%)	0.3074
Among working mothers	(*n*=50)	(*n*=81)	
I expect to lose my job soon due to COVID-19	4/50 (8.0%)	--	--
Part-time employment	36/50 (72.0%)	27/81 (33.3%)	**0.0004**
Currently working from home?	5/50 (10.0%)	4/81 (4.9%)	--
Afraid of COVID-19 in my workplace	26/50 (52.0%)	34/81 (42.0%)	0.1456
COVID-related stress scales	Mean (SD, range)	Mean (SD, range)	
COVID-19 impact scale (*n*=147)	1.03 (0.48, 0.0–2.3)	0.74 (0.44, 0.0–2.0)	**<0.0001**
COVID-19 anticipatory stigma Scale (*n*=130 early, 107 late)	1.75 (0.57, 1.0–3.8)	1.48 (0.42, 1–2.8)	**<0.0001**
Fears of contamination scale	2.14 (0.73, 1.0–4.0)	1.94 (0.69, 1.0–4.0)	**0.0001**
Fears of social distancing scale	2.59 (0.52, 1.0–4.0)	2.14 (0.55, 1.0–3.6)	**<0.0001**

For all scales, *n*=152 unless otherwise specified. *P*-value indicates significant difference between time points based on paired Wilcoxon signed-rank tests for continuous variables and McNemar paired test for discrete variables. Any measures not reported at follow-up were not asked at that time point. Bold *p*-values indicate significant differences over time

### COVID-19 Stress Scales: Impacts/Stigma/Fears

Distributions of reported COVID-19 impact, stigma, and fears are shown in Table 2 and Supplementary Figure 1. Since the 2020 Lockdown period, there was a significant mean decrease in the overall COVID-19 impact scale (1.03 at 2020 lockdown vs 0.74 at 1-year follow-up, possible range 0–3). Responses to components of each of the scales are shown in Supplementary Tables 1, 2, and 3. At both time points, the highest reported component of the impact scale was “change to routines,” followed by “a change to family income/employment” (Supplementary Table 1). At 2020 lockdown, a high proportion of women also reported either moderate or severe change to food access (24%), medical care access (37%), and loss of social support (34.8%). Significant reductions were seen across all individual items in the impact scale at 1-year follow-up relative to 2020 lockdown, with the exception of impact on mental healthcare access and family discord, which both remained relatively low over time.

The overall level of reported anticipatory stigma, and each item within the scale, also reduced significantly over time (Table 2 and Supplementary Table 2). At both time points, the highest stigma item reported was the worry that if a woman got COVID-19, she would not be able to take care of her children (≥82% at both time points). During the 2020 lockdown, nearly a quarter of the women worried at least some of the time that they would be deported if they got COVID-19, and a third worried that a family member would be deported if they got COVID-19, but this reduced to 8% and 14%, respectively, at follow-up. At 2020 lockdown, 57.7% worried at least some of the time they would lose their children if they got COVID, which reduced to 42.9% at follow-up. For every other item in the anticipatory stigma scale, at least half the participants reported worrying about it at least some of the time in 2020 lockdown, with significantly reduced levels later in the pandemic (Supplementary Table 2).

Fear levels related to contamination and social distancing at 2020 lockdown were similar, and both sets of fears reduced significantly later in COVID-19 (Table 2). The highest reported contamination fear at both time points was the fear that a family member may get sick or die, followed nearly equally by fears that the mother herself would “get a bad virus,” “get very sick,” or “have to go to a hospital” and that “people in the world may get sick or die.” The highest social distancing fears at both time points were the fears that “it will be hard to do things I like,” “I am afraid I will miss a lot of work,” and “I am afraid I will be stuck at home.” All items in both fear scales reduced significantly over time (Supplementary Table 3).

### Correlations with Anxiety and Depressive Symptoms

At both time points, all COVID-19-related stressors/fears significantly correlated with each other and with higher levels of anxiety and depressive symptoms (Supplementary Tables 4 and 5). Also at both time points, acculturation negatively correlated with the anticipatory stigma scale. Individual items of the impact scale were nearly all correlated with depression, and all correlated with anxiety at both time points (Supplementary Tables 6 and 7). COVID-19 impact on stress was the item most highly correlated with depression and anxiety at both time points.

We found positive bivariate correlations of changes in COVID-19 stressors over time with changes in anxiety and depression over time (Supplementary Table 5). For example, a change (∆) in stigma, impact, and fear of contamination were all moderately correlated with a change (∆) in anxiety (*r*=0.28, 0.23, 0.17, respectively; all *p*<0.05), while a change in depression was moderately correlated with a change in COVID-19 impact (*r*=0.30, *p*=0.0002) and fear of social distancing over time (*r*=0.20, *p*=0.0015).

### Linear Regressions with Anxiety and Depressive Symptoms at Each Time Point

Results of regression models indicated that each scale of anticipatory stigma, impact, and fears individually associated with higher levels of anxiety and depressive symptoms at both time points, after adjusting for maternal age, relationship status, education, and acculturation (Table 3). All of these associations remained significant after Bonferroni corrections. To examine COVID-19 impact further, we assessed how each item within the impact scale associated with anxiety and depressive symptoms. We found at the 2020 lockdown period, nearly all items significantly associated with greater anxiety and depressive symptoms, while at follow-up, all items remained associated significantly with greater anxiety, and most items associated with greater depression, even after Bonferroni correction, with the exception of changes to routine, loss of income, and loss of food access (Supplementary Table 8).

**Table 3:** Regression results predicting anxiety and depressive symptoms

	Anxiety (STAI)		Depressive symptoms (CES-D)	
	Estimate (SE)	*p*-values	Estimate (SE)	*p*-values
2020 lockdown period (*n*= 117–139)				
Anticipatory stigma	2.32 (0.54)	<0.0001*	2.62 (0.66)	0.0001*
COVID-19 impact scale	3.68 (0.54)	<0.0001*	4.03 (0.68)	<0.0001*
Fear contamination	2.12 (0.39)	<0.0001*	2.36 (0.48)	<0.0001*
Fear social distancing	2.35 (0.55)	<0.0001*	3.26 (0.66)	<0.0001*
1-year follow-up (*n*=96–135)				
Anticipatory stigma	3.66 (0.85)	<0.0001*	3.47 (1.06)	0.0015*
COVID-19 impact scale	3.39 (0.86)	0.0002*	3.72 (1.03)	0.0005*
Fear contamination	1.37 (0.45)	0.0029*	2.08 (0.54)	0.0002*
Fear social distancing	1.82 (0.57)	0.0018*	2.96 (0.68)	<0.0001*

All models are adjusted for covariates of maternal age, relationship status, education, and acculturation. Significant associations are shown in bold, and those that pass Bonferroni correction for the eight tests at each time point (*p*<0.006) are additionally starred

### Linear Regressions with Change Over Time

On average, stigma, impact and fears all decreased over time, as did depression and anxiety symptoms from 2020 to 1-year follow-up (Tables 1 and 2). When we analyzed change in COVID-19 stressors in relation to anxiety and depression at follow-up, it did not reveal any significant associations. However, models of *change* in COVID-19 stressors with *change* in anxiety and depression revealed significant associations. Specifically, changes in anticipatory stigma, COVID-19 impact, and fear of contamination were all positively associated with changes in anxiety. Change in COVID-19 impact and fear of social distancing both positively associated with changes in levels of depression over time (Table 4). The largest effect was for change in COVID-19 impact on change in depression: (B=3.06, SE=0.84; *p*=0.0004). This was the only association with the change scores that remained significant after Bonferroni correction.

**Table 4:** Regression results predicting changes in anxiety and depressive symptoms

	Change in anxiety (STAI)		Change in depressive symptoms (CES-D)	
	Estimate (SE)	*p* values	Estimate (SE)	*p* values
Change in anticipatory stigma (*n*=81)	2.142 (0.822)	**0.011**	1.160 (0.922)	0.212
Change in COVID-19 impact scale (*n*=133)	1.825 (0.688)	**0.0089**	3.059 (0.842)	0.0004*
Change in fear contamination (*n*=133)	1.106 (0.483)	**0.024**	0.788 (0.617)	0.204
Change in fear social distancing	0.804 (0.601)	0.184	1.756 (0.743)	**0.0196**

All models are adjusted for covariates of maternal age, relationship status, education, and acculturation. Significant associations are shown in bold, and those that pass Bonferroni correction for the eight tests (*p*<0.006) are additionally starred. Change in COVID-19 stigma, impact, and fears, depression, and anxiety are calculated as levels at 1-year follow-up minus levels at 2020 lockdown

## Discussion

We examined the socioeconomic and mental health effects of the pandemic on mothers of Mexican-descent at two time points across the first year of the pandemic in Southern California. Overall, we found a striking socioeconomic impact early in the pandemic, such that many of the women reported job loss or reduced working hours. This socioeconomic impact was reduced later in the pandemic as quarantines were lifted and more women were employed and working outside the home. The rate of employment at 1-year follow-up (53.3%) was in fact similar to the rate (51%) when women were initially recruited in the study (2013–2019), implying full recovery to pre-pandemic rates. However, a consistently high proportion of women continued to report significant socioeconomic impacts later in the pandemic, e.g., about a quarter reported the primary income earner of their household lost their job at both time points. We also discovered a decrease in depression and anxiety symptoms across the first year of the pandemic, but levels were still higher than pre-pandemic levels, as reported elsewhere in this sample [34].

Women reported relatively high levels of overall COVID-19 impact, anticipatory stigma, and fears of contamination and of social distancing early in the pandemic. These findings are in line with expectations of cultural stress theory, which posits exacerbated stress for families who face high levels of stigma and discrimination, with subsequent impacts on mental health. The fact that a quarter of our participants feared deportation, and a third feared deportation of a family member at 2020 lockdown highlights the heavy burden of cultural stressors faced by Latina mothers in the pandemic. Despite reduced levels at 1-year follow-up, these COVID-19-related stressors were all associated with increased levels of anxiety and depressive symptoms at both time points, after adjusting for demographics and acculturation level. These findings highlight the urgency of the COVID-19 impact on mental health, which necessitates continued social policies to alleviate impacts (e.g., child tax credits), along with increased mental health screening and treatment as the pandemic continues.

The significant socioeconomic impact of the pandemic on Latina families in our sample was expected as Latina women experienced the highest rates of job loss nationally early in the pandemic [6]. A similar study among Latina mothers early in the pandemic found that even the receipt of the stimulus payment did not sufficiently relieve perceived economic pressures or reduce worries, anxiety, or depression symptoms [35]. Similar to our findings of increased and lasting income insecurity and food insecurity, US Census data have shown 37% of Latinx families in California (32.8% in the USA) report somewhat or very difficult to pay for usual household expenses a year into the pandemic (August 2021) and 11% of Californian (10% of USA) Latinx families report food insufficiency at least some of the time [36].

The impact of the pandemic on all domains of Latina mothers’ lives was severe. Extreme changes to routine and family income and employment were expected, given the immediate impact of quarantine on daily lives and jobs. Further, we saw a dramatic and immediate impact on food access, medical and mental healthcare access, and social support. While reports of impact on all of these factors significantly decreased a year later in the pandemic, there remained a relatively high number of women reporting moderate or severe change to these factors. Additionally, many women reported fears of deportation if they were diagnosed with COVID-19, or of losing their children, adding another layer of stress for immigrant groups. While these fears reduced over time, there were still many women who feared at least some of the time that they (8.3%) or their family members (14.1%) could get deported later in the pandemic. The fear of losing their children if they were to get COVID-19 was among one of the highest fears reported at both time points, second only to not being able to take care of their children. This fear is a significant burden on Latina women and requires better communication during future pandemics to ensure that all immigrant women are seeking care and getting vaccinated without fear of losing their children.

All of these COVID-19 impact factors were associated with increased levels of anxiety and depressive symptoms at both time points. Notably, these associations were already evident during the 2020 lockdown period, even at a time when only one woman in the sample tested positive for COVID-19. Later in the pandemic, over a quarter of the women reported testing positive, though COVID-19 impacts, stigmas, and fears were all reduced, likely as more information was gained and disseminated to the public. These findings indicate that anticipatory fears, perhaps even greater than realized fears, can have important psychological effects, similar to other studies in the USA [35] and in China [37, 38]. Additionally, we found a longitudinal trend that changes in COVID-19-related stressors associated with changes in anxiety and depression over time, with the largest effect for changes to overall COVID-19 impact positively associated with significant changes to levels of depression over time. These findings highlight the need for urgent and immediate attention to Latina women’s mental health during a time of global crisis.

### Recommendations

Based on the severe socioeconomic and mental health impacts of the pandemic on Latina mothers, we provide some clinical, public health, and policy recommendations. Clinicians should assess women’s mental health during wellness visits and provide recommendations for ways to reduce depressive symptoms and anxiety. Clinicians can also provide referrals to Spanish-speaking mental health specialists. Public health departments can provide resources to expand access to mental healthcare in low-income and immigrant neighborhoods. Greater educational resources or Spanish-language public health campaigns, particularly through social media, could be used to assure immigrant Latina women of their privacy and safety when seeking out medical care or vaccinations [21]. To address the ongoing socioeconomic impacts of the pandemic, government assistance programs could be expanded to ease food and income insecurity, particularly to enhance access for immigrant communities. These programs have provided supplemental nutrition for low-income families, regardless of immigration status, but are historically underutilized by Latinx immigrant parents [39]. School counselors could educate parents about these resources and reassure families that applications will not put their families at deportation risk. Income assistance could also be expanded for immigrant families during times of crisis, as the CARES ACT provided recovery rebates to most low-income US households, but excluded non-citizens and their US citizen children from benefiting.

### Limitations

Our results should be considered in light of several limitations. Our data represent a relatively small sample of mothers of Mexican descent living in Southern California across the first year of the pandemic. Southern California may be unique as a border region with a long history of immigrants integrated into the community, but also a strong presence of immigration control and a high cost of living, relative to other regions. We also only surveyed mothers, though they are arguably the most vulnerable in the family, as Mexican American mothers tend to take on the bulk of household labor [40], while often still working and managing homeschooling during the pandemic. Additionally, all measures were self-reported, and the data were drawn from only two time points across the first year of the pandemic. We also note that our study had a low response rate of 49%. However, this response is typical in studies of vulnerable populations, who are particularly under high stress during a pandemic. Thus, the women in our study may underrepresent the most vulnerable women in the study, who were less likely to participate.

## Conclusions

Our findings indicate a severe impact of the COVID-19 pandemic across all domains of Mexican-descent mothers’ lives, including economic, social, and family. COVID-19 impacts, anticipatory stigma, and fears all associated with worse psychological health both early and later in the pandemic. While COVID-19 impacts diminished over time, they remained associated with elevated anxiety and depression at 1-year follow-up, indicating that the long duration of the pandemic has lasting deleterious effects on mental health of Latina mothers. In line with cultural stress theory, we found that Latina mothers who entered the pandemic with socioeconomic disadvantages also faced stigmas and fears related to their marginalized status that increased the mental health burden on Latina mothers and restricted them from seeking care. As the pandemic continues into its third year, these findings highlight the necessity of continued financial support as well as improving mental healthcare screening and access for these families, many of whom were already disadvantaged and with limited access to care prior to the pandemic [21].

## Supplementary information

ESM 1 (DOCX 93 kb)

## Data Availability

Data are available upon request to the authors.
