# Supplementary material for: COVID-19-Related Risk, Resilience, and Mental Health Among Mexican American Mothers Across the First Year of the Pandemic
Source: J Racial Ethn Health Disparities. 2023 Nov 8;12(1):49–58. doi: 10.1007/s40615-023-01849-2 (PMC11753345; doi:10.1007/s40615-023-01849-2)
Supplement: Supplementary file 1 — (DOCX 93 kb) [file 40615_2023_1849_MOESM1_ESM.docx]

Supplementary Table 1: Responses to items in the COVID-19 Impact Scale at 2020 Lockdown and 1 Year Follow-up

| COVID-19 impact items | Mean  (SD, range) | |  | No Change (0) | Mild  Change (1) | Moderate  Change (2) | Severe  Change (3) | p-value |
| --- | --- | --- | --- | --- | --- | --- | --- | --- |
| Rate how much the Coronavirus (COVID-19) pandemic has changed your life in each of the following ways, including change to: | | | | | | | | |
| 1. Routines | |  |  |  |  |  |  | **<0.0001** |
| 2020 Lockdown | | 2.03  (0.84, 0-3) |  | 7/152 (4.6%) | 30/152 (19.7%) | 66/152 (43.4%) | 49/152 (32.2%) |  |
| 1 Year Follow-up | | 1.57  (0.97, 0-3) |  | 23/147 (15.6%) | 44/147 (29.9%) | 53/147 (36.1%) | 27/147 (18.4%) |  |
| 2. Family income/employment | |  |  |  |  |  |  | **<0.0001** |
| 2020 Lockdown | | 1.51  (0.90, 0-3) |  | 25/152 (16.4%) | 42/152 (27.6%) | 68/152 (44.7%) | 17/152 (11.2%) |  |
| 1 Year Follow-up | | 1.04  (0.87, 0-3) |  | 47/147 (32.0%) | 52/147 (35.4%) | 43/147 (29.3%) | 5/147 (3.4%) |  |
| 3. Food access | |  |  |  |  |  |  | **<0.0001** |
| 2020 Lockdown | | 1.01  (0.76, 0-3) |  | 38/152 (25.0%) | 79/152 (52.0%) | 30/152 (19.7%) | 5/152 (3.3%) |  |
| 1 Year Follow-up | | 0.63  (0.72, 0-2) |  | 76/147 (51.7%) | 50/147 (34.0%) | 21/147 (14.3%) | 0/147 (0.0%) |  |
| 4. Medical care access | |  |  |  |  |  |  | **0.0053** |
| 2020 Lockdown | | 1.12  (0.80, 0-3) |  | 39/152 (25.7%) | 57/152 (37.5%) | 55/152 (36.2%) | 1/152 (0.66%) |  |
| 1 Year Follow-up | | 0.88  (0.88, 0-3) |  | 61/147 (41.5%) | 47/147 (32.0%) | 34/147 (23.1%) | 5/147 (3.4%) |  |
| 5. Mental health care access | |  | |  |  |  |  | 0.1423 |
| 2020 Lockdown | | 0.45  (0.78, 0-3) | | 107/152 (70.4%) | 24/152 (15.8%) | 18/152 (11.8%) | 3/152 (2.0%) |  |
| 1 Year Follow-up | | 0.37  (0.68, 0-3) | | 108/147 (73.5%) | 24/147 (16.3%) | 14/147 (9.5%) | 1/147 (0.7%) |  |
| 6. Social support access | |  | |  |  |  |  | **<0.0001** |
| 2020 Lockdown | | 1.22  (0.93. 0-3) | | 36/152 (23.7%) | 63/152 (41.4%) | 37/152 (24.3%) | 16/152 (10.5%) |  |
| 1 Year Follow-up | | 0.80  (0.83, 0-3) | | 64/147 (43.5%) | 53/147 (36.1%) | 26/147 (17.7%) | 4/147 (2.7%) |  |
| 7. Stress symptoms related to COVID-19 | |  | |  |  |  |  | **0.0003** |
| 2020 Lockdown | | 1.14  (0.76, 0-3) | | 33/152 (21.7%) | 65/152 (42.8%) | 53/152 (34.9%) | 1/152 (0.66%) |  |
| 1 Year Follow-up | | 0.90  (0.79, 0-3) | | 49/147 (33.3%) | 69/147 (46.9%) | 24/147 (16.3%) | 5/147 (3.4%) |  |
| 8. Family discord | |  | |  |  |  |  | 0.3736 |
| 2020 Lockdown | | 0.64  (0.71, 0-2) | | 75/152 (49.3%) | 56/152 (36.8%) | 21/152 (13.8%) | 0/152 (0%) |  |
| 1 Year Follow-up | | 0.61  (0.75, 0-3) | | 79/147 (53.7%) | 50/147 (34.0%) | 15/147 (10.2%) | 3/147 (2.0%) |  |

Sample size restricted to those not missing >-2 items in the total scale. P values calculated from paired Wilcox test.

Supplementary Table 2: Responses to items in the Anticipatory Stigma Scale

| **COVID-19 Stigma Scale items** | mean  (sd, range**)** | Not worried at all (1)  n (%) | Worried some of the time (2)  n (%) | Worried most of  the time (3)  n (%) | Worried all of the time (4)  n (%) | p-value |
| --- | --- | --- | --- | --- | --- | --- |
| Do you worry that if you get COVID-19 … | | | | | | |
| - - 1. You will be treated differently by family members outside your household? |  |  |  |  |  | **0.0495** |
| 2020 Lockdown | 1.74  (0.75, 1-4) | 54/130 (41.5%) | 60/130 (46.2%) | 12/130 (9.2%) | 4/130 (3.1%) |  |
| 1 Year Follow-up | 1.51  (0.72, 1-4) | 62/107 (57.9%) | 39/107 (36.4%) | 2/107 (1.9%) | 4/107 (3.7%) |  |
| - - 1. You will be excluded from family activities now and/or in the future? |  |  |  |  |  | **<0.0001** |
| 2020 Lockdown | 1.91  (0.76, 1-4) | 38/129 (29.5%) | 71/129 (55.0%) | 14/129 (10.9%) | 6/129 (4.7%) |  |
| 1 Year Follow-up | 1.51  (0.69, 1-4) | 62/107 (57.9%) | 37/107 (34.6%) | 6/107 (5.6%) | 2/107 (1.9%) |  |
| - - 1. You will be excluded from social gatherings now and/or in the future |  |  |  |  |  | **0.0024** |
| 2020 Lockdown | 1.81  (0.83, 1-4) | 49/129 (38.0%) | 64/129 (49.6%) | 7/129 (5.4%) | 9/129 (7.0%) |  |
| 1 Year Follow-up | 1.49  (0.65, 1-4) | 62/107 (57.9%) | 40/107 (37.4%) | 3/107 (2.8%) | 2/107 (1.9%) |  |
| - - 1. You will be excluded from religious activities now and/or in the future |  |  |  |  |  | **<0.0001** |
| 2020 Lockdown | 1.60  (0.72, 1-4) | 67/130 (51.5%) | 51/130 (39.2%) | 9/130 (6.9%) | 3/130 (2.3%) |  |
| 1 Year Follow-up | 1.27  (0.47, 1-3) | 79/107 (73.8%) | 27/107 (25.2%) | 1/107 (0.9%) | 0/107 (0%) |  |
| - - 1. You will be deported |  |  |  |  |  | **0.0007** |
| 2020 Lockdown | 1.29  (0.6, 1-4) | 100/129 (77.5%) | 23/129 (17.8%) | 4/129 (3.1%) | 2/129 (1.6%) |  |
| 1 Year Follow-up | 1.11  (0.42, 1-4) | 98/107 (91.6%) | 7/107 (6.5%) | 1/107 (0.9%) | 1/107 (0.9%) |  |
| - - 1. If a family member gets COVID-19 , they will be deported? |  |  |  |  |  | **0.0003** |
| 2020 Lockdown | 1.38  (0.60, 1-4) | 87/130 (66.9%) | 37/130 (28.5%) | 5/130 (3.8%) | 1/130 (0.8%) |  |
| 1 Year Follow-up | 1.18  (0.49, 1-4) | 91/106 (85.8%) | 12/106 (11.3%) | 2/106 (1.9%) | 1/106 (0.9%) |  |
| - - 1. You will lose your children |  |  |  |  |  | **0.0311** |
| 2020 Lockdown | 1.88  (0.97, 1-4) | 55/130 (42.3%) | 49/130 (37.7%) | 12/130 (9.2%) | 14/130 (10.8%) |  |
| 1 Year Follow-up | 1.58  (0.81, 1-4) | 60/105 (57.1%) | 34/105 (32.4%) | 6/105 (5.7%) | 5/105 (4.8%) |  |
| - - 1. You will lose healthcare benefits? |  |  |  |  |  | **0.0143** |
| 2020 Lockdown | 1.56  (0.81, 1-4) | 77/129 (59.7%) | 38/129 (29.5%) | 8/129 (6.2%) | 6/129 (4.7%) |  |
| 1 Year Follow-up | 1.29  (0.55, 1-4) | 80/107 (74.8%) | 24/107 (22.4%) | 2/107 (1.9%) | 1/107 (0.9%) |  |
| - - 1. You will not be able to find a job in the future? |  |  |  |  |  | **0.0079** |
| 2020 Lockdown | 1.69  (0.76, 1-4) | 61/130 (46.9%) | 50/130 (38.5%) | 17/130 (13.1%) | 2/130 (1.5%) |  |
| 1 Year Follow-up | 1.47  (0.70, 1-4) | 68/107 (63.6%) | 30/107 (28.0%) | 7/107 (6.5%) | 2/107 (1.9%) |  |
| - - 1. You will not be able to take care of your children? |  |  |  |  |  | **0.0108** |
| 2020 Lockdown | 2.65  (0.99, 1-4) | 15/130 (11.5%) | 49/130 (37.7%) | 33/130 (25.4%) | 33/130 (25.4%) |  |
| 1 Year Follow-up | 2.36  (0.99, 1-4) | 19/107 (17.8%) | 52/107 (48.6%) | 15/107 (14.0%) | 21/107 (19.6%) |  |

Sample size restricted to those not missing >-2 items in the total scale. P values calculated from paired Wilcox test.

Supplementary Table 3. Table Responses to items in the COVID-19 Fears of Illness and Virus Evaluation Scale

| **COVID-19 Fears Scale items** | mean  (sd, range**)** | Not afraid at all (1)  n (%) | Afraid some of the time (2)  n (%) | Afraid most of  the time (3)  n (%) | Afraid all of the time (4)  n (%) | p-value |
| --- | --- | --- | --- | --- | --- | --- |
| **Fears of contamination Scale** |  |  |  |  |  |  |
| How often have you felt afraid or worried about the following item in the last week? | | | | | |  |
| 1. I am afraid I may get a bad illness or virus. |  |  |  |  |  | **0.0012** |
| 2020 Lockdown | 2.25  (0.89, 1-4) | 26/151 (17.2%) | 82/151 (54.3%) | 23/151 (15.2%) | 20/151 (13.2%) |  |
| 1 Year Follow-up | 2.01  (0.78, 1-4) | 35/150  (23.3%) | 87/150  (58.0%) | 19/150  (12.7%) | 9/150  (6.0%) |  |
| 1. I am afraid I will get very, very sick if I catch a bad illness or virus. |  |  |  |  |  | **0.0005** |
| 2020 Lockdown | 2.26  (0.96, 1-4) | 29 /151 (19.2%) | 78/151 (51.7%) | 19/151 (12.6%) | 25/151 (16.6%) |  |
| 1 Year Follow-up | 2.0  (0.77, 1-4) | 35/150  (23.3%) | 89/150  (59.3%) | 17/150  (11.3%) | 9/150  (6.0%) |  |
| 1. I am afraid I will have to go to the hospital because of a bad illness or virus. |  |  |  |  |  | **0.0014** |
| 2020 Lockdown | 2.28  (0.95, 1-4) | 29/151  (19.2%) | 74/151  (49.0%) | 25/151  (16.6%) | 23/151  (15.2%) |  |
| 1 Year Follow-up | 2.03  (0.90, 1-4) | 44/150  (29.3%) | 72/150  (48.0%) | 20/150  (13.3%) | 14/150  (9.3%) |  |
| 1. I am afraid I might die if I get a bad illness or virus. |  |  |  |  |  | **0.0007** |
| 2020 Lockdown | 2.20  (0.99, 1-4) | 38/151  (25.2%) | 68/151  (45.0%) | 22/151  (14.6%) | 23/151  (15.2%) |  |
| 1 Year Follow-up | 1.94  (0.90, 1-4) | 52/149  (34.9%) | 67/149  (45.0%) | 17/149  (11.4%) | 13/149  (8.7%) |  |
| 1. I am afraid my pet might get a bad illness or virus. |  |  |  |  |  | **0.1585** |
| 2020 Lockdown | 1.25  (0.62, 1-4) | 124/151  (82.1%) | 20/151  (13.2%) | 3/151  (2.0%) | 4/151  (2.6%) |  |
| 1 Year Follow-up | 1.34  (0.70, 1-4) | 113/150  (75.3%) | 29/150  (19.3%) | 2/150  (1.3%) | 6/150  (4.0%) |  |
| 1. I am afraid a family member might get sick or die because of a bad illness or virus. |  |  |  |  |  | **<0.0001** |
| 2020 Lockdown | 2.53  (0.99, 1-4) | 21/151  (13.9%) | 63/151  (41.7%) | 33/151  (21.9%) | 34/151  (22.5%) |  |
| 1 Year Follow-up | 2.21  (0.92, 1-4) | 31/150  (20.7%) | 77/150  (51.3%) | 22/150  (14.7%) | 20/150  (13.3%) |  |
| 1. I am afraid I may do something that would cause someone else to get a bad illness or virus. |  |  |  |  |  | **0.0011** |
| 2020 Lockdown | 2.14  (0.88, 1-4) | 36/151  (23.8%) | 71/151  (47.0%) | 31/151  (20.5%) | 13/151  (8.6%) |  |
| 1 Year Follow-up | 1.91  (0.83, 1-4) | 49/150  (32.7%) | 76/150  (50.7%) | 15/150  (10.0%) | 10/150  (6.7%) |  |
| 1. I am afraid a friend might get sick or die because of a bad illness or virus |  |  |  |  |  | **0.0037** |
| 2020 Lockdown | 2.14  (0.85, 1-4) | 30/151  (19.9%) | 85/151  (56.3%) | 21/151  (13.9%) | 15/151  (9.9%) |  |
| 1 Year Follow-up | 1.93  (0.84, 1-4) | 48/150  (32.0%) | 75/150  (50.0%) | 17/150  (11.3%) | 10/150  (6.7%) |  |
| 1. I am afraid people in the world might get sick or die because of a bad illness or virus. |  |  |  |  |  | **0.0180** |
| 2020 Lockdown | 2.25  (0.85, 1-4) | 23/151  (15.2%) | 84/151  (55.6%) | 27/151  (17.9%) | 17/151  (11.3%) |  |
| 1 Year Follow-up | 2.07  (0.85, 1-4) | 36/150  (24.0%) | 80/150  (53.3%) | 21/150  (14.0%) | 13/150  (8.7%) |  |
| **Fears of Social Distancing** |  |  |  |  |  |  |
| How often have you felt afraid or worried about the following item in the last week? | | | | | |  |
| 1. I am afraid I will be stuck at home because of a bad illness or virus. |  |  |  |  |  | **0.0016** |
| 2020 Lockdown | 2.11  (0.93, 1-4) | 41/151  (27.2%) | 68/151  (45.0%) | 26/151  (17.2%) | 16/151  (10.6%) |  |
| 1 Year Follow-up | 1.86  (0.83 1-4) | 54/150  (36.0%) | 72/150  (48.0%) | 15/150  (10.0%) | 9/150  (6.0%) |  |
| 1. I am afraid it will be hard to do things I like because of a bad illness or virus. |  |  |  |  |  | **0.0012** |
| 2020 Lockdown | 2.17  (0.83, 1-4) | 27/151  (17.9%) | 86/151  (57.0%) | 24/151  (15.9%) | 14/151  (9.3%) |  |
| 1 Year Follow-up | 1.90  (0.83, 1-4) | 51/150  (34.0%) | 72/150  (48.0%) | 18/150  (12.0%) | 9/150  (6.0%) |  |
| 1. I am afraid I will miss a lot of work because of a bad illness or virus. |  |  |  |  |  | **<0.0001** |
| 2020 Lockdown | 2.15  (0.96, 1-4) | 41/151  (27.2%) | 64/151  (42.4%) | 28/151  (18.5%) | 18/151  (11.9%) |  |
| 1 Year Follow-up | 1.78  (0.88, 1-4) | 67/150  (44.7%) | 55/150  (36.7%) | 17/150  (11.3%) | 9/150  (6.0%) |  |
| 1. I am afraid I will not be able to see friends (for a long time) because of a bad illness or virus. |  |  |  |  |  | **0.0001** |
| 2020 Lockdown | 2.03  (0.92, 1-4) | 47/151  (31.1%) | 68/151  (45.0%) | 21/151  (13.9%) | 15/151  (9.9%) |  |
| 1 Year Follow-up | 1.72  (0.80, 1-4) | 68/150  (45.3%) | 63/150  (42.0%) | 12/150  (8.0%) | 7/150  (4.7%) |  |
| 1. I am afraid I will be sad and lonely because of a bad illness or virus. |  |  |  |  |  | **0.0021** |
| 2020 Lockdown | 1.91  (0.88, 1-4) | 54/151  (35.8%) | 69/151  (45.7%) | 16/151  (10.6%) | 12/151  (7.9%) |  |
| 1 Year Follow-up | 1.68  (0.82, 1-4) | 75/150  (50.0%) | 55/150  (36.7%) | 13/150  (8.7%) | 7/150  (4.7%) |  |

Sample size restricted to those not missing >-2 items in the total scale. P values calculated from paired Wilcox test.

Bold indicates significant change from early to 1 Year Follow-up at p<0.05.

Supplementary Table 4: Bivariate correlations of primary study variables at 2020 Lockdown.

|  | Stigma | Impact | Fear of Contam. | Fear of Soc. Dist. | SASH | CES-D | STAI |
| --- | --- | --- | --- | --- | --- | --- | --- |
| Stigma | 1 |  |  |  |  |  |  |
| Impact | **0.49*** | 1 |  |  |  |  |  |
| Fear of Contam. | **0.47*** | **0.42*** | 1 |  |  |  |  |
| Fear of Soc. Dist | **0.44*** | **0.44*** | **0.71*** | 1 |  |  |  |
| SASH | **-0.25** | *-0.14* | 0.09 | 0.08 | 1 |  |  |
| CES-D | **0.24** | **0.37*** | **0.36*** | **0.35*** | **0.30*** | 1 |  |
| STAI | **0.30** | **0.48*** | **0.38*** | **0.32*** | 0.02 | **0.63*** | **1** |

Bold indicates significant correlations at p<0.05, italics indicates marginal at p< 0.1 *Indicates significant after Bonferroni correction for 36 tests in the table (<0.0014). Contam.=Contamination; Soc. Dist.=Social Distancing; SASH=Short Acculturation Scale for Hispanics; CES-D=Center for Epidemiologic Studies Depression Scale; STAI=State Trait Anxiety Inventory.

Supplementary Table 5: Bivariate correlations of primary study variables at 1 year Follow-up.

|  | Stigma | Impact | Fear of Contam. | Fear of Soc. Dist. | ∆ Stigma | ∆ Impact | ∆ Fear of Contam. | ∆ Fear of Soc. Dist. | SASH | CES-D | STAI | ∆ CES-D | ∆  STAI |
| --- | --- | --- | --- | --- | --- | --- | --- | --- | --- | --- | --- | --- | --- |
| Stigma | 1 |  |  |  |  |  |  |  |  |  |  |  |  |
| Impact | **0.53*** | 1 |  |  |  |  |  |  |  |  |  |  |  |
| Fear of Contam. | **0.58*** | **0.33*** | 1 |  |  |  |  |  |  |  |  |  |  |
| Fear of Soc. Dist. | **0.52*** | **0.38*** | **0.68*** | 1 |  |  |  |  |  |  |  |  |  |
| ∆ Stigma | **0.33** | 0.03 | 0.16 | 0.10 | 1 |  |  |  |  |  |  |  |  |
| ∆ Impact | 0.09 | **0.50*** | 0.05 | 0.13 | **0.17** | 1 |  |  |  |  |  |  |  |
| ∆ Fear of Contam. | **0.30** | 0.02 | **0.43*** | **0.23** | **0.33** | **0.18** | 1 |  |  |  |  |  |  |
| ∆ Fear of Soc. Dist. | **0.21** | 0.10 | **0.27** | **0.58*** | *0.19* | **0.23** | **0.50*** | 1 |  |  |  |  |  |
| SASH | *-0.19* | -0.12 | 0.07 | 0.08 | 0.01 | 0.00 | -0.04 | -0.03 | 1 |  |  |  |  |
| CES-D | **0.28** | **0.34*** | **0.33*** | **0.37*** | 0.00 | *0.15* | -0.02 | 0.11 | **0.23** | 1 |  |  |  |
| STAI | **0.41*** | **0.39*** | **0.26** | **0.30*** | 0.09 | 0.07 | -0.06 | 0.02 | -0.05 | **0.65*** | 1 |  |  |
| ∆ CES-D | 0.10 | 0.12 | 0.08 | 0.13 | 0.17 | **0.30*** | 0.09 | **0.20** | -0.06 | **0.53*** | **0.27** | 1 |  |
| ∆ STAI | 0.15 | 0.08 | 0.07 | 0.12 | **0.28** | **0.23** | **0.17** | *0.15* | -0.05 | **0.32*** | **0.57*** | **0.51*** | 1 |

Bold indicates significant correlations at p<0.05, italics indicates marginal at p< 0.1 *Indicates significant after Bonferroni correction for 105 tests in the table (<0.0005). Contam.=Contamination; Soc. Dist.=Social Distancing; SASH=Short Acculturation Scale for Hispanics; CES-D=Center for Epidemiologic Studies Depression Scale; STAI=State Trait Anxiety Inventory; All variables listed are measured at 1 Year Follow-up, unless marked with ∆, indicating the change in measure over time (1 Year Follow-up – 2020 Lockdown time point).

Supplementary Table 6: Bivariate correlations with each COVID-19 Impact item and other key variables at 2020 Lockdown

|  | Routines | Income | Food  Access | Medical Care access | Mental health access | Social support | Stress | Family discord | Personal diagnosis | SASH | CES-D | STAI | COVID-19 Impact Score |
| --- | --- | --- | --- | --- | --- | --- | --- | --- | --- | --- | --- | --- | --- |
| Routines | 1 |  |  |  |  |  |  |  |  |  |  |  |  |
| Income | **0.39*** | 1 |  |  |  |  |  |  |  |  |  |  |  |
| Food access | **0.30*** | **0.54*** | 1 |  |  |  |  |  |  |  |  |  |  |
| Medical access | **0.27*** | **0.17** | **0.31*** | 1 |  |  |  |  |  |  |  |  |  |
| Mental health access | **0.20** | **0.23*** | **0.34*** | **0.41*** | 1 |  |  |  |  |  |  |  |  |
| Social support | **0.40*** | **0.33*** | **0.44*** | **0.33*** | **0.40*** | 1 |  |  |  |  |  |  |  |
| Stress | **0.42*** | **0.38*** | **0.42*** | **0.22** | **0.26*** | **0.47*** | 1 |  |  |  |  |  |  |
| Family discord | **0.36*** | **0.29*** | **0.29*** | **0.21** | **0.38*** | **0.42*** | **0.40*** | 1 |  |  |  |  |  |
| Personal diagnosis | 0.05 | 0.11 | 0.11 | **0.20** | **0.28*** | 0.10 | 0.11 | **0.23*** | 1 |  |  |  |  |
| SASH | 0.07 | *-0.14* | **-0.19** | **-0.21** | **-0.19** | -0.09 | 0.03 | 0.03 | -0.01 | 1 |  |  |  |
| CES-D | **0.30*** | 0.10 | *0.16* | 0.03 | **0.33*** | **0.25** | **0.44*** | **0.35*** | 0.08 | **0.30*** | 1 |  |  |
| STAI | **0.33*** | **0.21** | **0.38*** | **0.25*** | **0.37*** | **0.31** | **0.41*** | **0.26*** | *0.14* | 0.02 | **0.63*** | 1 |  |
| COVID-19 Impact Score | **0.64*** | **0.64*** | **0.68*** | **0.56*** | **0.62*** | **0.73*** | **0.67*** | **0.64*** | **0.31*** | *-0.14* | **0.37*** | **0.48*** | 1 |

Bold indicates significant correlations at p<0.05, italics indicates marginal at p< 0.1. *Indicates significant after Bonferroni correction for 105 tests in the table (p<0.0005). SASH=Short Acculturation Scale for Hispanics; CES-D=Center for Epidemiologic Studies Depression Scale; STAI=State Trait Anxiety Inventory.

Supplementary Table 7: Bivariate correlations with each COVID-19 Impact item and other key variables at 1 Year-Follow-up

|  | Routines | Income | Food Access | Medical Care Access | Mental Health Access | Social Supports | Stress | Family Discord | Personal Diagnosis | SASH | CESD-D | STAI | COVID-19 Impact Score |
| --- | --- | --- | --- | --- | --- | --- | --- | --- | --- | --- | --- | --- | --- |
| Routines | 1 |  |  |  |  |  |  |  |  |  |  |  |  |
| Income | **0.23** | 1 |  |  |  |  |  |  |  |  |  |  |  |
| Food Access | *0.16* | **0.59*** | 1 |  |  |  |  |  |  |  |  |  |  |
| Medical Care Access | **0.26** | **0.39*** | **0.36*** | 1 |  |  |  |  |  |  |  |  |  |
| Mental Health Access | 0.13 | **0.34*** | **0.33*** | **0.42*** | 1 |  |  |  |  |  |  |  |  |
| Social Supports | **0.34*** | **0.31*** | **0.28** | **0.48*** | **0.49*** | 1 |  |  |  |  |  |  |  |
| Stress | **0.38*** | **0.29*** | 0.12 | **0.38*** | **0.37*** | **0.41*** | 1 |  |  |  |  |  |  |
| Family Discord | **0.23** | **0.31*** | **0.32*** | **0.46*** | **0.35*** | **0.38*** | **0.60*** | 1 |  |  |  |  |  |
| Personal Diagnosis | 0.08 | -0.08 | -0.08 | 0.03 | -0.05 | -0.05 | 0.07 | -0.02 | 1 |  |  |  |  |
| SASH | 0.11 | -0.13 | **-0.18** | **-0.20** | -0.11 | *-0.14* | 0.03 | -0.03 | 0.06 | 1 |  |  |  |
| CESD-D | 0.14 | 0.11 | *0.16* | **0.17** | **0.24** | **0.22** | **0.42*** | **0.37*** | 0.09 | **0.23** | 1 |  |  |
| STAI | **0.21** | **0.23** | **0.20** | **0.28** | **0.26** | **0.21** | **0.36*** | **0.33*** | 0.00 | -0.05 | **0.65*** | 1 |  |
| COVID-19 Impact Score | **0.55*** | **0.66*** | **0.58*** | **0.72*** | **0.64*** | **0.70*** | **0.67*** | **0.67*** | 0.11 | -0.12 | **0.34*** | **0.39*** | 1 |

Bold indicates significant correlations at p<0.05, italics indicates marginal at p< 0.1. *Indicates significant after Bonferroni correction for 105 tests in the table (p<0.0005). SASH=Short Acculturation Scale for Hispanics; CES-D=Center for Epidemiologic Studies Depression Scale; STAI=State Trait Anxiety Inventory.

Supplementary Table 8: Regression results predicting depressive symptoms (Box-Cox transformed).

|  | Depressive Symptoms (CES-D) Transformed (Box-Cox) | | |
| --- | --- | --- | --- |
|  | Estimate (SE) | p values | |
| **2020 Lockdown Period**  **(n’s 117-139)** | | |  |
| Anticipatory Stigma | 0.98 (0.24) | **<0.0001*** | |
| COVID-19 Impact Scale | 1.40 (0.25) | **<0.0001*** | |
| Fear Contamination | 0.79 (0.16) | **<0.0001*** | |
| Fear Social Distancing | 1.04 (0.23) | **<0.0001*** | |
| **1 Year Follow-up**  **(n’s=96-135)** | | |  |
| Anticipatory Stigma | 1.07 (0.27) | **0.0002*** | |
| COVID-19 Impact Scale | 0.97 (0.21) | **<0.0001*** | |
| Fear Contamination | 0.58 (0.15) | **0.0002*** | |
| Fear Social Distancing | 0.83 (0.19) | **<0.0001*** | |

All models are adjusted for covariates of maternal age, relationship status, education, and acculturation. Significant associations are shown in bold, and those that pass Bonferroni correction for the eight tests at each time point (p<0.006) are additionally starred. Models of depression were Box-Cox transformed (after adding 1) because the residuals of the models with depression were not normally distributed.

Supplementary Table 9: COVID-19 Impact items associate with anxiety and depressive symptoms at both 2020 Lockdown and 1 Year Follow-up.

|  | **Anxiety**  **(n=139 2020 Lockdown; 135 1 year follow-up)** | | **Depression**  **(n=139 2020 Lockdown; 134 1 year follow-up)** | |
| --- | --- | --- | --- | --- |
| COVID-19 Impact Scale Item | Estimate (std error) | p values | Estimate (std error) | p values |
| Change to routine |  |  |  |  |
| 2020 Lockdown | 1.45 (0.33) | **<0.0001*** | 1.64 (0.42) | **0.0001*** |
| 1 Year Follow-up | 0.75 (0.34) | **0.0310** | 0.52 (0.42) | 0.2214 |
| Loss of income |  |  |  |  |
| 2020 Lockdown | 1.02 (0.33) | **0.0024*** | 0.85 (0.41) | **0.0399** |
| 1 Year Follow-up | 0.87 (0.38) | **0.0229** | 0.52 (0.47) | 0.2278 |
| Loss of food access |  |  |  |  |
| 2020 Lockdown | 1.94 (0.36) | **<0.0001*** | 1.36 (0.48) | **0.0049** |
| 1 Year Follow-up | 0.93 (0.46) | **0.0426** | 1.20 (0.56) | **0.0341** |
| Loss of medical healthcare access |  |  |  |  |
| 2020 Lockdown | 1.11 (0.37) | **0.0028*** | 0.61 (0.46) | 0.1887 |
| 1 Year Follow-up | 1.35 (0.35) | **0.0002*** | 1.43 (0.44) | **0.0016*** |
| Loss of mental healthcare access |  |  |  |  |
| 2020 Lockdown | 1.80 (0.36) | **<0.0001*** | 2.32 (0.44) | **<0.0001*** |
| 1 Year Follow-up | 1.44 (0.48) | **0.0031** | 1.85 (0.59) | **0.0021*** |
| Loss of social support |  |  |  |  |
| 2020 Lockdown | 1.25 (0.32) | **0.0001*** | 1.58 (0.39) | **<0.0001*** |
| 1 Year Follow-up | 0.92 (0.39) | **0.0198** | 1.48 (0.47) | **0.0021*** |
| COVID-19-related Stress |  |  |  |  |
| 2020 Lockdown | 2.05 (0.35) | **<0.0001*** | 2.76 (0.42) | **<0.0001*** |
| 1 Year Follow-up | 1.70 (0.39) | **<0.0001*** | 2.50 (0.46) | **<0.0001*** |
| Increased family discord |  |  |  |  |
| 2020 Lockdown | 1.29 (0.40) | **0.0018*** | 2.13 (0.48) | **<0.0001*** |
| 1 Year Follow-up | 1.74 (0.41) | **<0.0001*** | 2.37 (0.50) | **<0.0001*** |

All models are adjusted for covariates of maternal age, relationship status, education, and acculturation. Significant associations are bolded, and those that pass Bonferroni correction for the 16 tests at each time point (p<0.003) are additionally starred.

Supplementary Figure 1: Histograms of COVID-19 stressors at 2020 Lockdown and 1 Year Follow-up.
